# Supplementary material for: Conjugated linoleic acid as a novel insecticide targeting the agricultural pest Leptinotarsa decemlineata
Source: PLoS One. 2019 Nov 14;14(11):e0220830. doi: 10.1371/journal.pone.0220830 (PMC6855466; doi:10.1371/journal.pone.0220830)

**Supplementary Figure S1. Greenhouse spray.** Foliar application with 16% aqueous CLA spray. Blue paper is an indicator strip that changes from yellow to blue after contact with water, indicating complete coverage of spray.


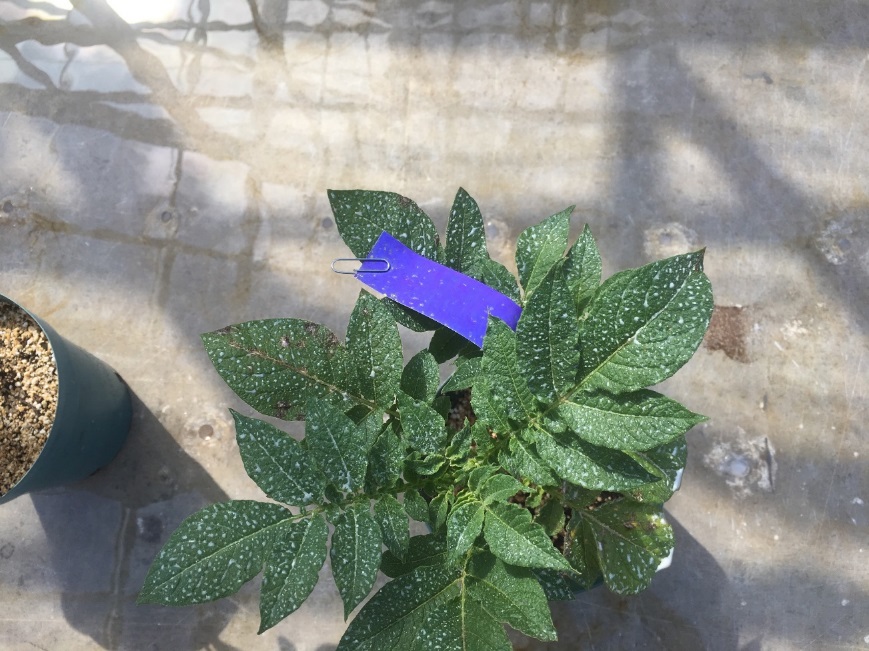

Supplement: S1 File — (DOCX) [file pone.0220830.s001.docx]
